# Serum Proteomic Analysis by Tandem Mass Tag-Based Quantitative Proteomics in Pediatric Obstructive Sleep Apnea

**DOI:** 10.3389/fmolb.2022.762336

**Published:** 2022-04-11

**Authors:** Hanrong Cheng, Shoumei Jin, Simin Huang, Tianyong Hu, Miao Zhao, Dongcai Li, Benqing Wu

**Affiliations:** ^1^ Institute of Respiratory Diseases, Shenzhen People’s Hospital, The Second Clinical Medical College of Jinan University, The First Affiliated Hospital of Southern University of Science and Technology, Shenzhen, China; ^2^ Longgang ENT Hospital, Institute of ENT and Shenzhen Key Laboratory of ENT, Shenzhen, China; ^3^ Department of Neonatology, University of Chinese Academy of Science-Shenzhen Hospital, Shenzhen, China

**Keywords:** obstructive sleep apnea, proteomic analysis, tandem mass tags, differentially expressed proteins, serum, experimental validation

## Abstract

Pediatric obstructive sleep apnea (OSA) is a frequent respiratory disorder with an estimated prevalence of 3–6% in the general population. However, the underlying pathophysiology of OSA remains unclear. Recently, proteomic analysis using high-resolution and high-throughput mass spectrometry has been widely used in the field of medical sciences. In the present study, tandem mass tag (TMT)-based proteomic analysis was performed in the serum of patients with OSA. The proteomic analysis revealed a set of differentially expressed proteins that may be associated with the pathophysiology of OSA. The differentially expressed proteins in patients with OSA were enriched in pathways including phagosome and glycan synthesis/degradation, immune response, and the hedgehog signaling pathway, indicating that such functions are key targets of OSA. Moreover, the experimental validation studies revealed that four proteins including ANTXR1, COLEC10, NCAM1, and VNN1 were reduced in the serum from patients with moderate and severe OSA, while MAN1A1 and CSPG4 protein levels were elevated in the serum from patients with severe OSA. The protein levels of ANTXR1, COLEC10, NCAM1, and VNN1 were inversely correlated with apnea-hypopnea index (AHI) in the recruited subjects, while the protein level of MAN1A1 was positively correlated with AHI, and no significant correlation was detected between CSPG4 protein and AHI. In summary, the present study for the first time identified differentially expressed proteins in the serum from OSA patients with different severities by using TMT-based proteomic analysis. The functional enrichment studies suggested that several signaling pathways may be associated with the pathophysiology of OSA. The experimental validation results indicated that six proteins including ANTXR1, COLEC10, NCAM1, VNN1, CGPG4, and MAN1A1 may play important roles in the pathophysiology of OSA, which requires further mechanistic investigation.

## Introduction

Pediatric obstructive sleep apnea (OSA) is a type of sleep disorder and is featured by habitual snoring and repeated upper airway obstruction in children (Jordan et al., 2014) and affects about 3% of prepubertal children (Gozal et al., 2009). OSA has been suggested to be linked with cardiovascular and metabolic disorders such as insulin resistance and abnormal lipid metabolism (Lévy et al., 2015; Stöberl et al., 2017). Although habitual snoring is the common clinical characteristic of OSA patients, snoring is not associated with the high incidence of cardiovascular diseases. Various studies have been performed to decipher the pathogenesis of OSA by assessing the altered levels of potential serum biomarkers associated with oxidative stress and inflammation (Nadeem et al., 2013; Demirci Sağlam et al., 2019; Ji et al., 2021). Thus, it is important to further explore the serum features of OSA patients, which may be helpful to establish novel strategies for the better management of OSA. However, due to limitations in the traditional technologies, only a paucity of specific biomarkers for OSA has been detected. Therefore, novel approaches may be developed to provide new insights into the understanding of the pathophysiology of OSA.

Recently, proteomic analysis has been widely applied in the field of medical sciences. Two-dimensional electrophoresis (2-DE) coupled with mass spectrometry (MS) is commonly applied for proteomic analysis; however, this method has many drawbacks in protein identification; that is, proteins that are too large or small might be hard to recognize (Lee and Lee, 2004; Röcken et al., 2004; Mouzo et al., 2018). Moreover, extremely hydrophobic and low-abundance proteins are also difficult to recognize (Hanash, 2000). With the rapid progress in proteomic technologies, high-resolution and high-throughput mass spectrometry has become a mature technique. As one of the most robust proteomic techniques, tandem mass tags (TMTs) is a kind of relative and absolute quantitative technique of isotopic labeling. TMTs can identify and quantify proteins from multiple samples by high-resolution mass spectrometry series analysis at the same time (Rauniyar and Yates, 2014; Chahrour et al., 2015; Moulder et al., 2018). So far, TMTs have been widely used in the physiological and pathological research for animals and plants. Huang et al. used the TMT-based quantitative proteomic technique to identify potential serum diagnostic biomarkers for gastric cancer and successfully created the differentially expressed protein database of gastric cancer (Huang et al., 2018). TMT-based quantitative proteomic analysis revealed the deregulation of nicotinamide adenine dinucleotide metabolism and CD38 in inflammatory bowel disease (Ning et al., 2019). The TMT-based proteomic study revealed a panel of proteins differentially expressed in the follicular fluid of polycystic ovary syndrome (PCOS), and inflammatory, immunological, and metabolic abnormalities were identified inside the intra-follicular environment, which could be aggravated by obesity (Zhang et al., 2019a). In the field of OSA studies, Kohli et al. used isobaric tagging for the relative and absolute quantification (iTRAQ)-based proteomics approaches to identify differentially expressed proteins in adult OSA patients and found that the urinary endothelial protein c receptor (EPCR) and dermcidin may emerge as novel biomarkers for screening severe OSA patients (Kohli et al., 2019). Jurado-Gamez et al. used the iTRAQ techniques to study the serum proteomic changes in adults with OSA and provided initial evidence that differential protein expression occurs in adults with OSA and that such proteins change according to disease severity and appear to primarily involve lipid and vascular metabolic pathways (Jurado-Gamez et al., 2012). However, to our best knowledge, the TMT-based proteomic analysis has not been used to explore metabolomics profiling in OSA.

In this study, the TMT-based proteomic approach was used to investigate the differentially expressed proteins in the serum from pediatric OSA patients, aiming to reveal the differences in the proteomic profile. In addition, the biological significance of these differentially expressed proteins was uncovered to explore the mechanisms of pediatric OSA, which would be helpful to find out the underlying pathophysiology of pediatric OSA. The validation study was also performed to determine potential biochemical markers for pediatric OSA.

## Methods

### Ethical Statement

This study was approved by the Ethics Committee of Longgang ENT Hospital, Institute of ENT, and the Shenzhen Key Laboratory of ENT, and all parents of the children agreed and signed consent for this study.

### Recruitment of Patients and Sample Collection

A total of 64 children were included in this study. Overnight polysomnography was performed in all children. All testing was performed in the Sleep Physiology Laboratory in Longgang ENT Hospital, Institute of ENT, and the Shenzhen Key Laboratory of ENT with continuous observation of the child by a polysomnography technician skilled in pediatric polysomnography. A normal polysomnogram was defined by an apnea-hypopnea index (AHI) <1. Mild OSA was defined by an AHI of 1–5. Moderate OSA was defined by an AHI of 6–10. Severe OSAS was defined by an AHI >10 ^20^. The inclusion criteria for OSA patients were as follows: 1) 2–13 years old and 2) AHI > 1; the inclusion criteria for control subjects were as follows: 1) 2–13 years old and 2) AHI < 1. The exclusion criteria were as follows: 1) do not meet the inclusion criteria and 2) the presence of a known genetic syndrome or any other chronic disease. The clinical characteristics of the all the subjects are shown in Table 1. After overnight polysomnography, 5 ml of blood was collected in the morning by using a serum separator tube. The serum samples were allowed to clot at room temperature for 2 h before centrifugation at 1,000 g for 15 min. The collected serum samples were stored at –80 °C before downstream assays.

**TABLE 1:** Clinical characteristics of all the recruited subjects.

	Non-OSA	Mild OSA	Moderate OSA	Severe OSA
	(*n* = 16)	(*n* = 16)	(*n* = 16)	(*n* = 16)
Age (years old)	5.9 (2–11)	4.8 (3–8)	5 .2 (2–13)	5.4 (2–9)
BMI (kg/m2)	16.2 (13.6–20.6)	16.8 (13-29.7)	16.3 (13.2–20.9)	17.2 (14.5–21.5)
Awake SpO2 (%)	98 (97–100)	98 (97–100)	98 (97–99)	97 (96–98)
AHI	0.62 (0.2–0.9)	3.9 (3.2–4.9)	6.9 (5.6–9.2)	25.9 (10.9–55.8)
SpO2 minimum (%)	92 (87–96)	90 (83–93)	83 (72–90)	76 (51–88)
SpO2 mean (%)	98 (96–100)	98 (96–100)	98 (96–99)	96 (92–98)

### Sample Preparation for TMT Analysis

The present study randomly selected 12 serum samples for TMT Quantitative Proteomics analysis. Among these 12 samples, three serum samples were from the non-OSA patients, three serum samples were from mild OSA patients, three serum samples were from moderated OSA patients, and three serum samples were from severe OSA patients. The clinical characteristics of the included subjects are shown in Supplemental Table S1.

### Protein Extraction and Digestion

The abundant serum proteins were depleted using High Select Top 14 spin columns (Thermo #A36370) following the manufacturer-provided protocol. Briefly, 10 μl of serum was applied to each column and incubated for 10 min with end-over-end rotation. Depleted samples were collected by centrifugation at 2000 g for 2 min. Ice-cold 100% trichloroacetic acid was added to 20% final concentration. The proteins were allowed to precipitate on ice for 60 min and then pelleted for 10 min at 20,000 g at 4°C. The pellets were washed twice with ice-cold acetone and allowed to dry at room temperature. Dry protein pellets were resuspended in 8 M urea and 50 mM ammonium bicarbonate (ambic). The proteins were quantified with a BCA Protein Assay Kit (Bio-Rad, United States). This was followed by sodium dodecyl sulfate–polyacrylamide gel electrophoresis (SDS-PAGE) and Coomassie Bright Blue staining to compare and analyze the protein expression consistency among samples. The poteins were reduced by the addition of dithiothreitol (DTT) to 100 mM and incubating in a boiled water bath for 30 min. The samples were then mixed with 200 µL of UA buffer (8 M urea with 150 mM Tris-HCl, pH 8.0), followed by centrifugation at 12,000 g for 15 min. After discarding the supernatant, the samples were incubated with 100 µL of indole-3-acetic acid (IAA) (50 mM IAA in UA) with agitation for 1 min, followed by incubation at room temperature in the dark for 30 min. After that, the samples were centrifuged at 12,000 g for 10 min. After discarding the supernatant, the samples were mixed with 100 µL of UA buffer, followed by centrifugation at 12,000 g for 10 min. After that, the samples were mixed with 100 µl of tetraethylammonium bromide buffer, followed by 14,000 g centrifugation for 10 min. After that, the samples were mixed with 40 µl of trypsin buffer with 1 min agitation, followed by 18 h incubation at 37 C. After centrifugation at 12,000 g for 10 min, the digested samples were acidified by the addition of a proper amount of 0.1% trifluoroacetic acid and were desalted c-18 Cartridge. The eluted peptides were dried in a centrifugal evaporator. After drying, the peptide samples were suspended on ice in 200 µL of the lysis buffer (4% SDS, 150 mM Tris-HCl, and 100 mM DTT, pH 7.8). The tissue was disrupted with agitation by a homogenizer and then immediately boiled for 5 min. The samples were further ultrasonicated and boiled again for another 5 min. The undissolved cellular debris was removed by centrifugation at 16,000 rpm for 15 min.

### TMT Labeling of Peptides and High-pH Reverse-Phase Fractionation

TMT reagents were used for labeling of peptides according to the manufacturer’s instructions (Thermo Fisher Scientific). Each aliquot (50 µg of peptide equivalent) was reacted with one tube of the TMT reagent. The sample labeling was as follows: Group A1, 128C; Group A2, 129N; Group A3, 129C; Group B1, 130C; Group B2, 130C; Group B3, 121N; Group C1, 131C; Group C2, 127N; Group C3, 132C; Group D1, 133N; Group D2, 133C; and Group D3, 134N. Equal amounts of TMT labeling peptides were mixed in each group, and then, HPRP (Pierce™ High-pH Reversed-Phase Peptide Fractionation Kit, Thermo Fisher, Waltham, MA, United States) was used to fractionate peptides after drying. The samples were eventually collected into 10 components. Each component of the peptides was stored at −80 C for liquid chromatography–mass spectrometry (LC-MS) analysis.

### LC-MS Analysis

The redissolved peptide solution was taken for LC-MS/MS analysis, and each fractional component of the sample was injected once for a total of 10 times for mass spectrometry analysis. The high-performance liquid chromatography liquid-phase system Easy-nLC 1,200 was used for separation. Buffer solutions: A, 0.1% formic acid solution, and B, 80% acetonitrile solution. The chromatographic column was balanced with 100% A solution. The sample was loaded onto the chromatographic column, that is, trap column (20 mm × 100 μm, 5 μm-C18, Dr. Maisch GmbH). Then, the C-18 column (75 μm × 150 mm, 5 μm-c-18, Dr. Maisch GmbH) was used for separation, and the velocity was at 300 nL/min. The relative liquid gradient was as follows: linear-gradient of liquid B, 2–5% for 0–2 min; linear gradient of liquid B, 5–28% for 2–71 min; linear gradient of liquid B, 28–40% for 71–79 min; linear gradient of liquid B, 40–100% for 79–81 min; and liquid B maintained at 90% for 81–90 min. After chromatographic separation, the peptides were analyzed using a Q-Exactive HF-X mass spectrometer (Thermo Scientific, Waltham, MA, United States) with the following parameters: analysis duration, 90 min; detection method, positive ion; and scanning range of parent ions, 300–1800 m/z. The mass charge ratios of polypeptides and polypeptide fragments were collected as follows: 20 fragment profiles (MS2 scan, HCD) were collected after each full scan. The resolution of the first-level mass spectrometry was 60,000 at m/z 200, the AGC target was 3e6, and the first-level maximum IT was 50 ms. The resolution of the second-level mass spectrometry was 45,000 at m/z 200, the AGC target was 1e5, and the second-level maximum IT was 50 ms. The MS2 activation type was HCD; the isolation window was 1.6 m/z, and the normalized collision energy was 30.

### Protein Database Searching and Analysis

The raw files produced from LC-MS/MS were imported into proteome discoverer software (version 2.4). The protein database (192,367 proteins) was sourced from uniport-uniprot-homo-192367-20200629.fasta. The search parameters were set as follows: Type, Reporter ion MS2; isobaric labels, TMTpro 16plex; Enzyme, Trypsin; Reporter mass tolerance, 0.005 Da; Max Missed cleavages, 2; Peptide Tolerance, 10 ppm; MS/MS Tolerance, 0.02 Da; Fix modifications, Carbamidomethyl (C); Variable modifications, Oxidation (M), Acetyl (Protein N-term), Deamidation (N,Q), TMTpro (K), TMTpro (N-term); Database, uniport-uniprot-homo-192367-20200629.fasta; Database pattern: Target-Reverse; and Percolator (FDR) ≤ 0.01; Razor and unique peptides were used for protein quantification.

### Bioinformatics Analysis

Perseus software and R statistical computing software were used to analyze the bioinformatics data. Differentially expressed proteins were screened with the cutoff of a ratio fold-change of >1.20 and *p*-values < 0.05. In our study, differentially expressed proteins were compared among different groups. Combining the comparative analysis of variance (ANOVA), *t*-test, and FDR (Benjamini–Hochberg), all qualitative and quantitative protein analysis results were obtained. Hierarchical clustering was adopted to categorize expression data together according to the protein level. Huge amounts of data are produced by mass spectrometry technology in proteomics, which represents all the biological processes of the organism. The aim of bioinformatics analysis was to find the source and mechanism for biological changes. UniProt Knowledgebase (UniProtKB)/Swiss-Prot, Gene Ontology (GO) enrichment, Kyoto Encyclopedia of Genes and Genomes (KEGG), and protein interaction network analysis were adopted.

### Enzyme-Linked Immunosorbent Assay

For the validation studies, serum samples from 64 subjects were used for the determination of protein levels. The protein levels of anthrax toxin receptor 1 (ANTXR1), collectin-10 (COLEC10), neural cell adhesion molecule 1 (NCAM1), vanin 1 (VNN1) and chondroitin sulfate proteoglycan 4 (CSPG4) in the serum samples were determined by the respective enzyme-linked immunosorbent assay (ELISA) kits from CUSABIO (Wuhan, China), and the protein level of mannosidase alpha class 1A member 1 (MAN1A1) in the serum samples was determined by Reddnot Biotech (Kelowna, Canada). In brief, 100 µl of serum samples was added into respective precoated ELISA plates. The plates were incubated for 2 h at 37°C. After removing the liquid from each well, the plates were incubated with 100 µl of biotin-antibody (1x) for 1 h at 37 °C. After washing three times, the plates were incubated with 100 µl of horseradish peroxidase-avidin (1x) 1 h at 37 °C. After washing five times, the plates were incubated with the 3,3′,5,5′-tetramethylbenzidine substrate for 30 min at 37 °C. Then, 50 μL of the stop solution was added to each well to stop the reaction. The optical density was detected at a 450 nm wavelength within 15 min. Then, the stop solution was added. A standard curve was constructed by plotting a graph of the absorbance of each reference standard against its corresponding levels and used to determine each of the protein levels.

### Statistical Analysis

The data for ELISA were presented as mean ± standard deviation. The data analysis was performed using GraphPad Prism Software (GraphPad Software 7.0, La Jolla, United States). Significant differences among different groups were analyzed using one-way ANOVA, followed by Bonferroni’s post-hoc tests. The correlation between AHI and protein levels was analyzed by Pearson correlation analysis. *p* < 0.05 was considered statistically significant.

## Results

### Identification of Proteins

In the study, we obtained 59,578 peptide spectrum matching (PSM) numbers and 8,544 unique peptides, which were mapped to 754 protein groups with 752 being quantified as proteins. The details of the identified proteins are shown in Supplemental Table S2.

### General Information of the TMT-Based Proteomic Analysis

**FIGURE 1:**
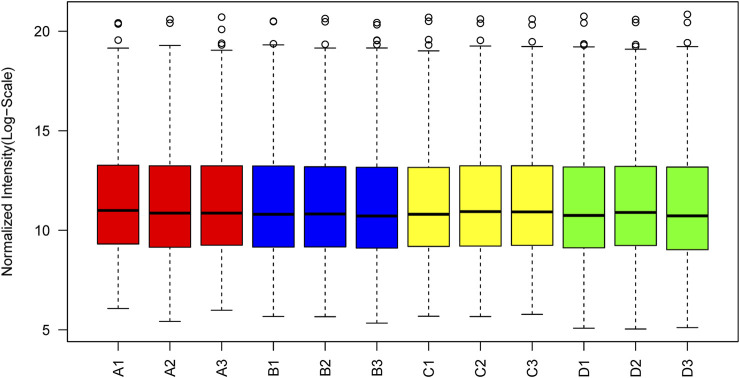
Box plots of log2 protein (or the reporter ion) intensity average for each sample used for TMT-based proteomic analysis.

**FIGURE 2:**
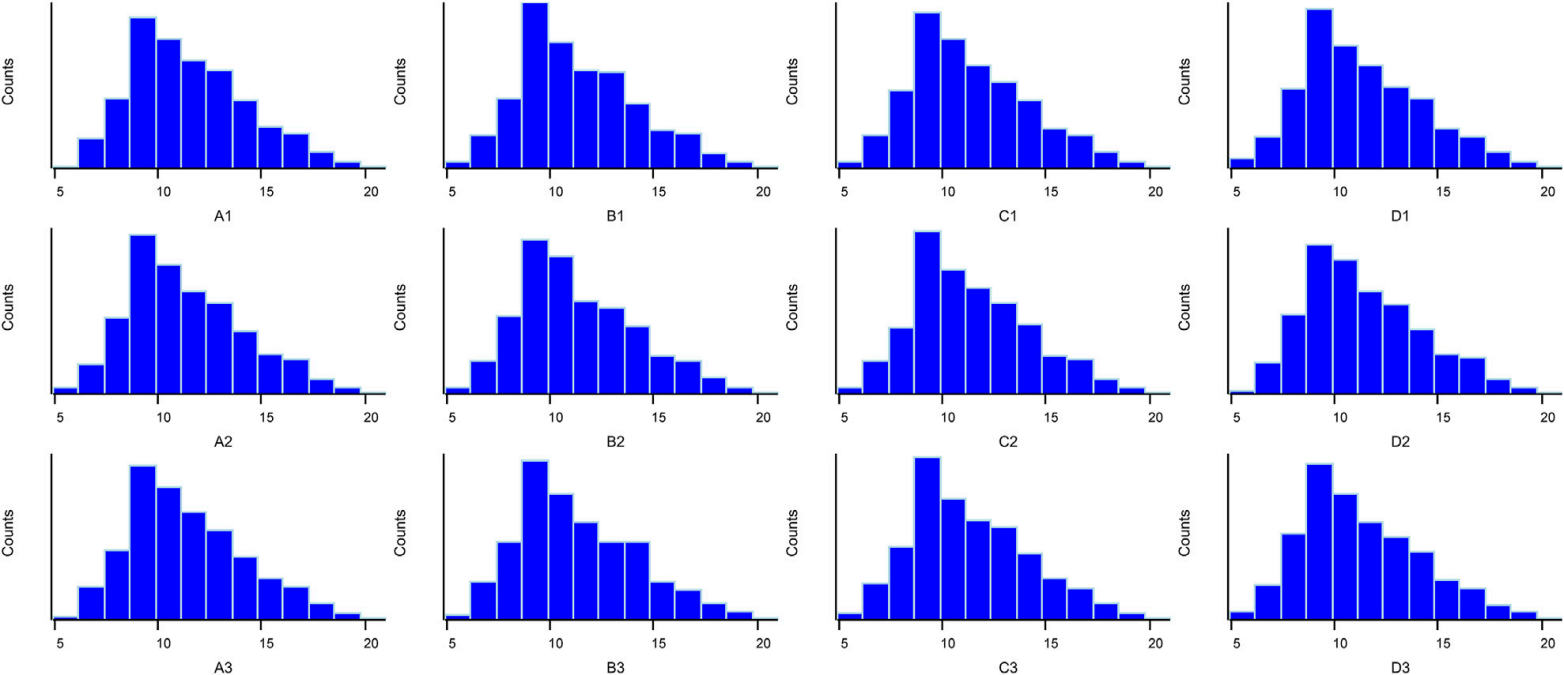
Histogram of log2 protein intensity for each serum sample in TMT-based proteomic analysis.

The box plot of normalized density (box plots of log2 protein intensity average for each sample used for TMT-based proteomic analysis) is shown in Figure 1. Figure 2 presents the histogram of intensity for each sample used for TMT-based proteomic analysis. In addition, the Pearson correlation coefficients of the samples exhibited high repeatability via assessment of relative quantitative proteins (Supplemental Figure S1).

### Hierarchical Cluster Analysis and Volcano Plots of Differentially Expressed Proteins

**FIGURE 3:**
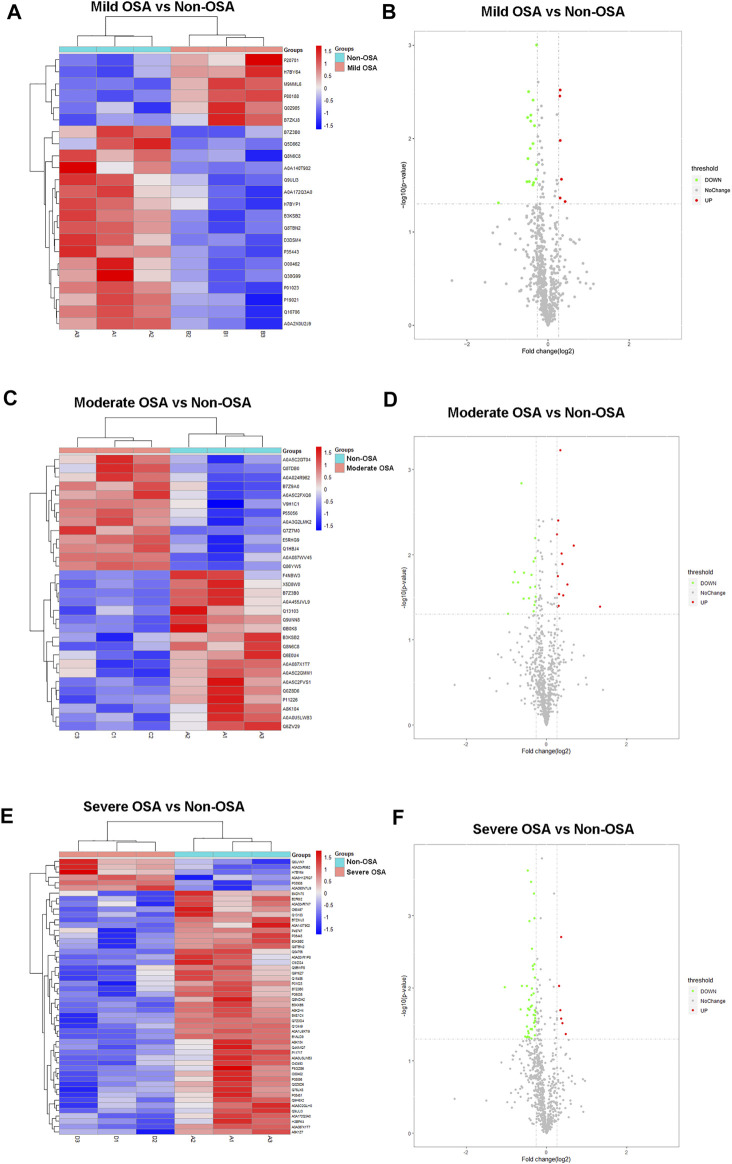
Hierarchical cluster analysis and volcano plots: **(A)** hierarchical cluster analysis and **(B)** volcano plot analysis of differentially expressed proteins between the mild OSA group and non-OSA group, **(C)** hierarchical cluster analysis and **(D)** volcano plot analysis of differentially expressed proteins between the moderate OSA group and non-OSA group, and **(E)** hierarchical cluster analysis and **(F)** volcano plot analysis of differentially expressed proteins between the severe OSA group and non-OSA group.

**FIGURE 4:**
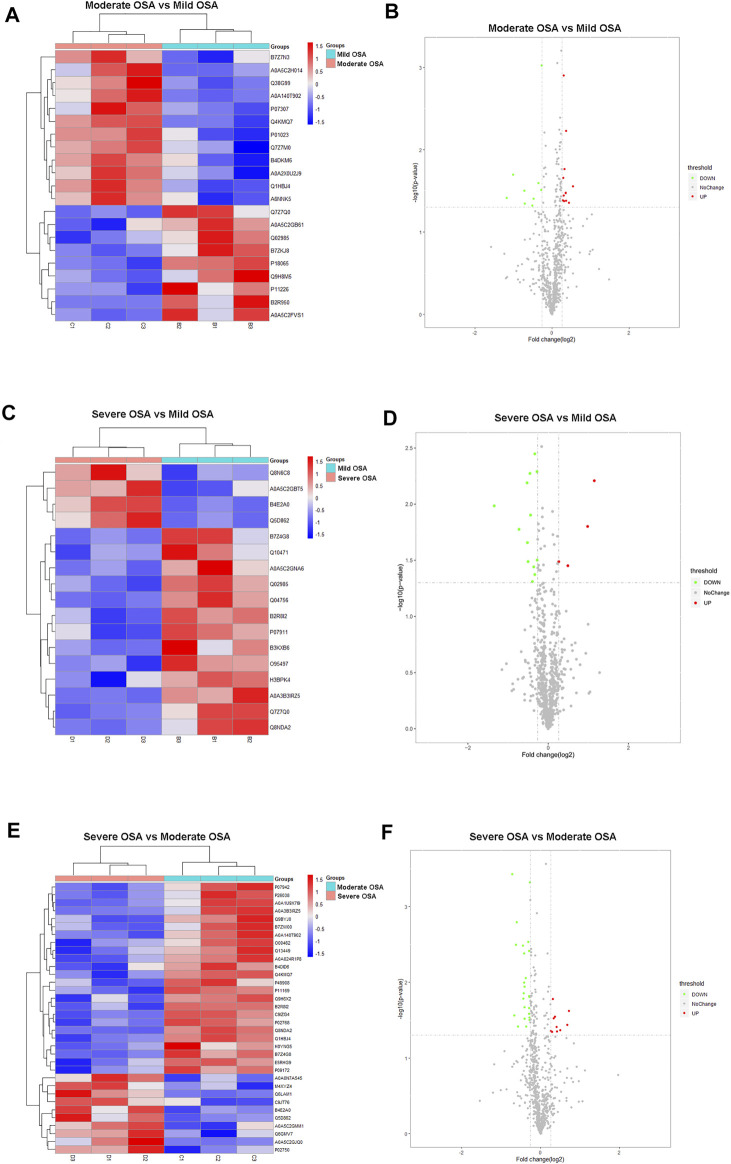
Hierarchical cluster analysis and volcano plots: **(A)** hierarchical cluster analysis and **(B)** volcano plot analysis of differentially expressed proteins between the moderate OSA group and mild OSA group, **(C)** hierarchical cluster analysis and **(D)** volcano plot analysis of differentially expressed proteins between the severe OSA group and mild OSA group, **(E)** hierarchical cluster analysis and **(F)** volcano plot analysis of differentially expressed proteins between the severe OSA group and moderate OSA group C.

The hierarchical cluster analysis and volcano plots of differentially expressed proteins between different groups are shown in Figures 3, 4. As shown in Figure 3, a total of 23 proteins (six upregulated and 17 downregulated) were significantly changed in the mild OSA group (Figures 3A,B), a total of 31 proteins (13 upregulated and 18 downregulated) were significantly changed in the moderate OSA group (Figures 3A,C,D), and a total of 53 proteins (four upregulated and 49 downregulated) were significantly changed in the severe group when compared to the non-OSA group (Figures 3E,F).

In addition, a total of 21 proteins (12 upregulated and nine downregulated) were significantly changed in the moderate OSA group (Figures 4A,B), and a total of 17 proteins (four upregulated and 13 downregulated) were significantly changed in the severe group when compared to the mild OSA group (Figures 4C,D). A total of 34 proteins (10 upregulated and 24 downregulated) were significantly changed in the severe group when compared to the moderate group (Figures 4E,F).

### GO Enrichment Analysis of Differentially Expressed Proteins

**FIGURE 5:**
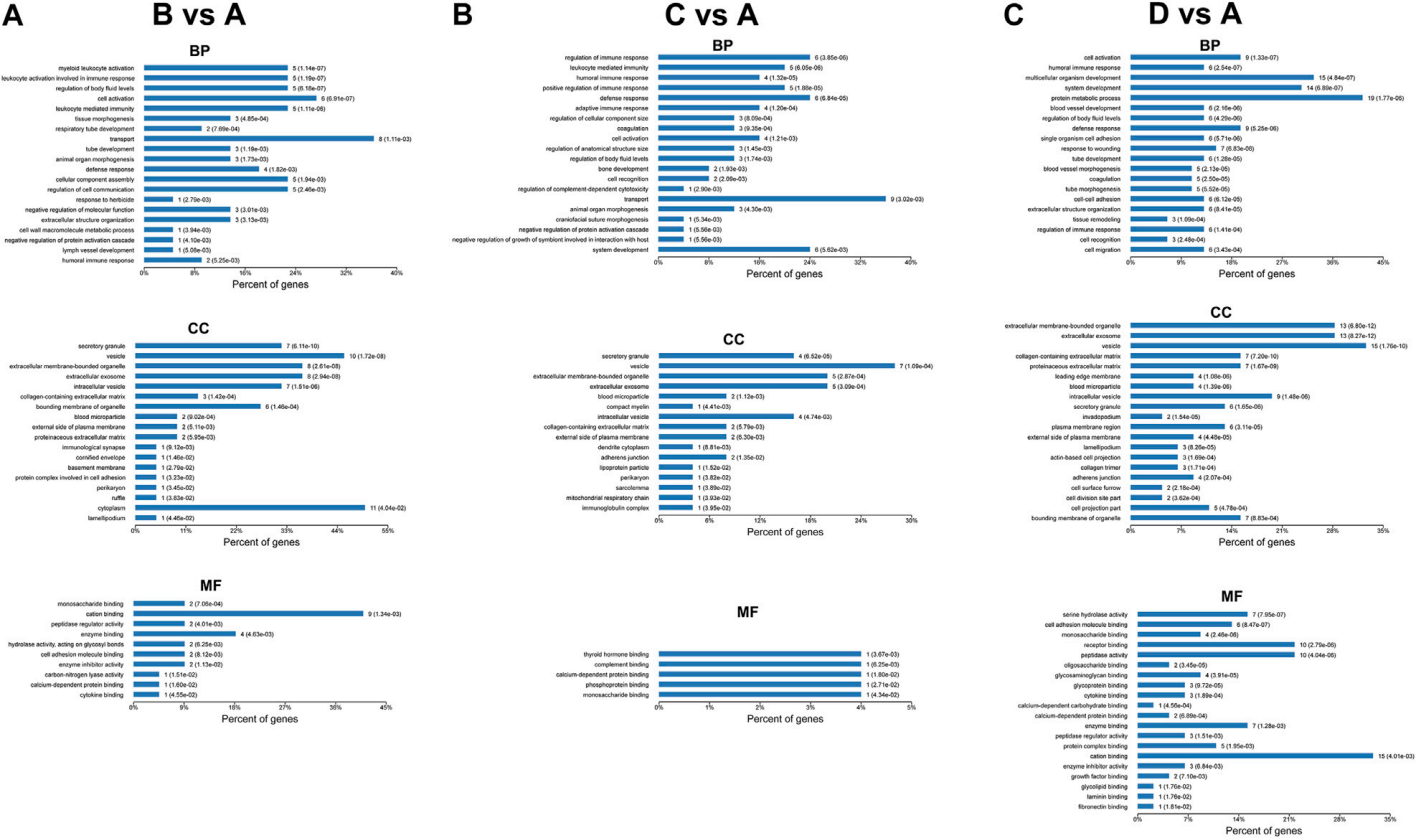
GO enrichment analysis of differentially expressed proteins: **(A)** group B (mild OSA) vs group A (non-OSA), **(B)** group C (moderate OSA) vs group A (non-OSA), and **(C)** group D (severe OSA) vs group A (non-OSA).

**FIGURE 6:**
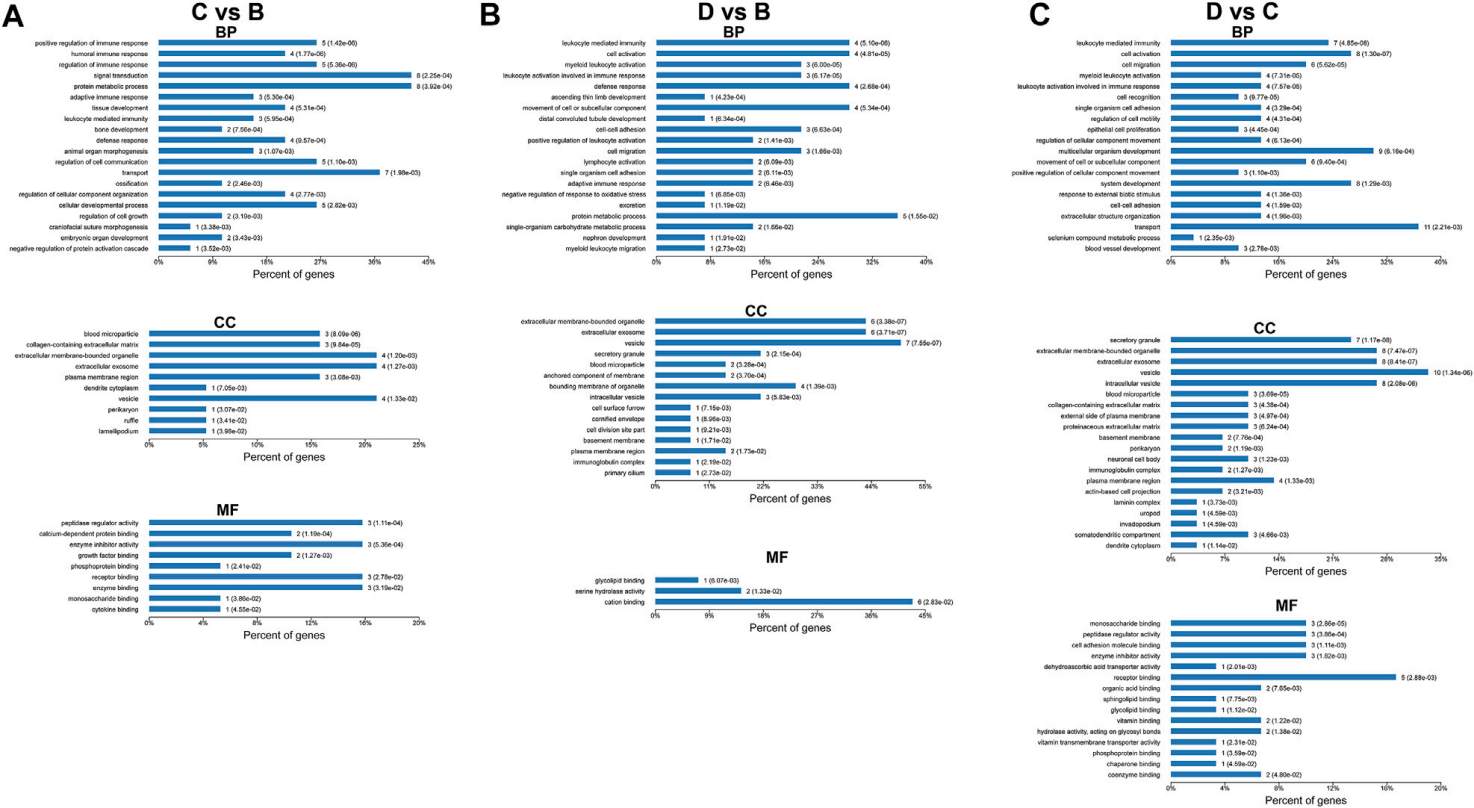
GO enrichment analysis of differentially expressed proteins: **(A)** group C (moderate OSA) vs group B (mild OSA), **(B)** group D (severe OSA) vs group B (mild OSA), and **(C)** group D (severe OSA) vs group C (moderate OSA).

The most enriched terms of GO enrichment analysis between different groups are shown in Figures 5, 6. For the differentially expressed proteins between mild OSA and non-OSA groups, proteins were mainly enriched in “myeloid leukocyte activation,” “leukocyte activation involved in the immune response,” “regulation of body fluid levels,” etc. under the category of biological process; were enriched in “secretory granule,” “vesicle,” “extracellular membrane-bounded organelle,” “intracellular vesicle,” etc. under the category of cellular component; and were enriched in “monosaccharide binding,” “cation binding,” “peptidase regulator activity,” etc. under the category of molecular function. For the differentially expressed proteins between moderate OSA and non-OSA groups, proteins were mainly enriched in “regulation of immune response,” “leukocyte mediated immunity,” “humoral immune response,” etc. under the category of biological process; were enriched in “secretory granule,” “vesicle,” “extracellular membrane-bounded organelle,” etc. under the category of cellular component; and were enriched in “thyroid hormone binding,” “complement binding,” “calcium-dependent protein binding,” etc. under the category of molecular function (Figure 5B). For the differentially expressed proteins between severe OSA and non-OSA groups, proteins were mainly enriched in “cell activation,” “humoral immune response,” “multicellular organism development,” etc. under the category of biological process; were enriched in “extracellular membrane-bounded organelle,” “extracellular exosome,” “vesicle,” etc. under the category of cellular component; and were enriched in “serine hydrolase activity,” “cell adhesion molecule binding,” “receptor binding,” etc. under the category of molecular function.

For the differentially expressed proteins between moderate and mild OSA groups, proteins were mainly enriched in “positive regulation of immune response,” “humoral immune response,” “regulation of immune response,” etc. under the category of biological process; were enriched in “blood microparticle,” “collagen-containing extracellular matrix,” “extracellular membrane-bounded organelle,” etc. under the category of cellular component; and were enriched in “peptidase regulator activity,” “calcium-dependent protein binding,” “enzyme inhibitor activity” etc. under the category of molecular function. For the differentially expressed proteins, between severe and mild OSA groups, proteins were mainly enriched in “leukocyte mediated immunity,” “cell activation,” and “myeloid leukocyte activation” under the category of biological process; were enriched in “extracellular membrane-bounded organelle,” “extracellular exosome,” “vesicle” etc. under the category of cellular component; and were mainly enriched in “glycolipid binding,” “serine hydrolase activity,” and “cation binding” under the category of molecular function. For the differentially expressed proteins between severe OSA and moderate OSA groups, proteins were mainly enriched in “leukocyte mediated immunity,’ “cell activation,” “cell migration,” etc. under the category of biological process; were mainly enriched in “secretory granule,” “extracellular membrane-bounded organelle,” “extracellular exosome,” etc. under the category of cellular component; and were mainly enriched in “monosaccharide binding,” “peptidase regulator activity”, “cell adhesion molecule binding,” etc. under the category of molecular function.

### KEGG Enrichment Analysis of Differentially Expressed Proteins

**FIGURE 7:**
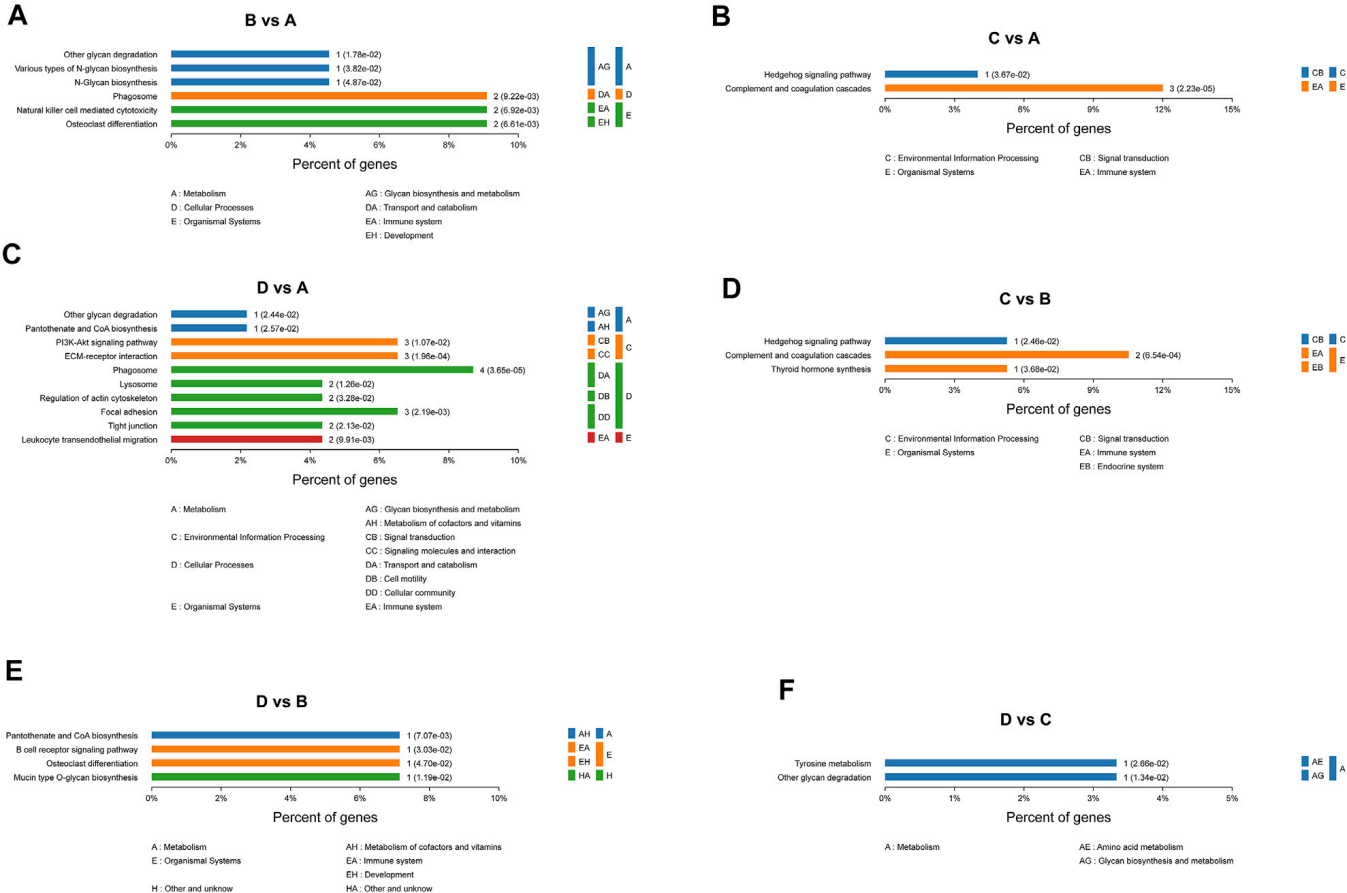
KEGG enrichment analysis of differentially expressed proteins: **(A)** group B (mild OSA) vs group A (non-OSA), **(B)** group C (moderate OSA) vs group A (non-OSA), **(C)** group D (severe OSA) vs group A (non-OSA), and **(D)** group C (moderate OSA) vs group B (mild OSA), and **(E)** group D (severe OSA) vs group B (mild OSA), and **(F)** group D (severe OSA) vs group C (moderate OSA).

The differentially expressed proteins between severe OSA and non-OSA groups were mainly enriched in “other glycan degradation,” “various types of N-glycan biosynthesis,” “N-glycan biosynthesis,” and so on (Figure 7A). The differentially expressed proteins between moderate OSA and non-OSA groups were mainly enriched in “Hedgehog signaling pathway” and “Complement and coagulation cascades” (Figure 7B). The differentially expressed proteins between severe OSA and non-OSA groups were mainly enriched in “other glycan degradation,” “pantothenate and CoA biosynthesis,” “PI3K-Akt signaling pathway,” and so on (Figure 7C). The differentially expressed proteins between moderate and mild OSA groups were mainly enriched in “Hedgehog signaling pathway,” “Complement and coagulation cascades,” and “Thyroid hormone synthesis” (Figure 7D). The differentially expressed proteins between severe OSA and mild OSA groups were mainly enriched in “Pantothenate and CoA biosynthesis,” “B cell receptor signaling pathway,” “Osteoclast differentiation,” and so on (Figure 7E). The differentially expressed proteins between severe OSA and moderate OSA groups were mainly enriched in “Tyrosine metabolism” and “Other glycan degradation” (Figure 7F).

### PPI Network Analysis of Differentially Expressed Proteins

**FIGURE 8:**
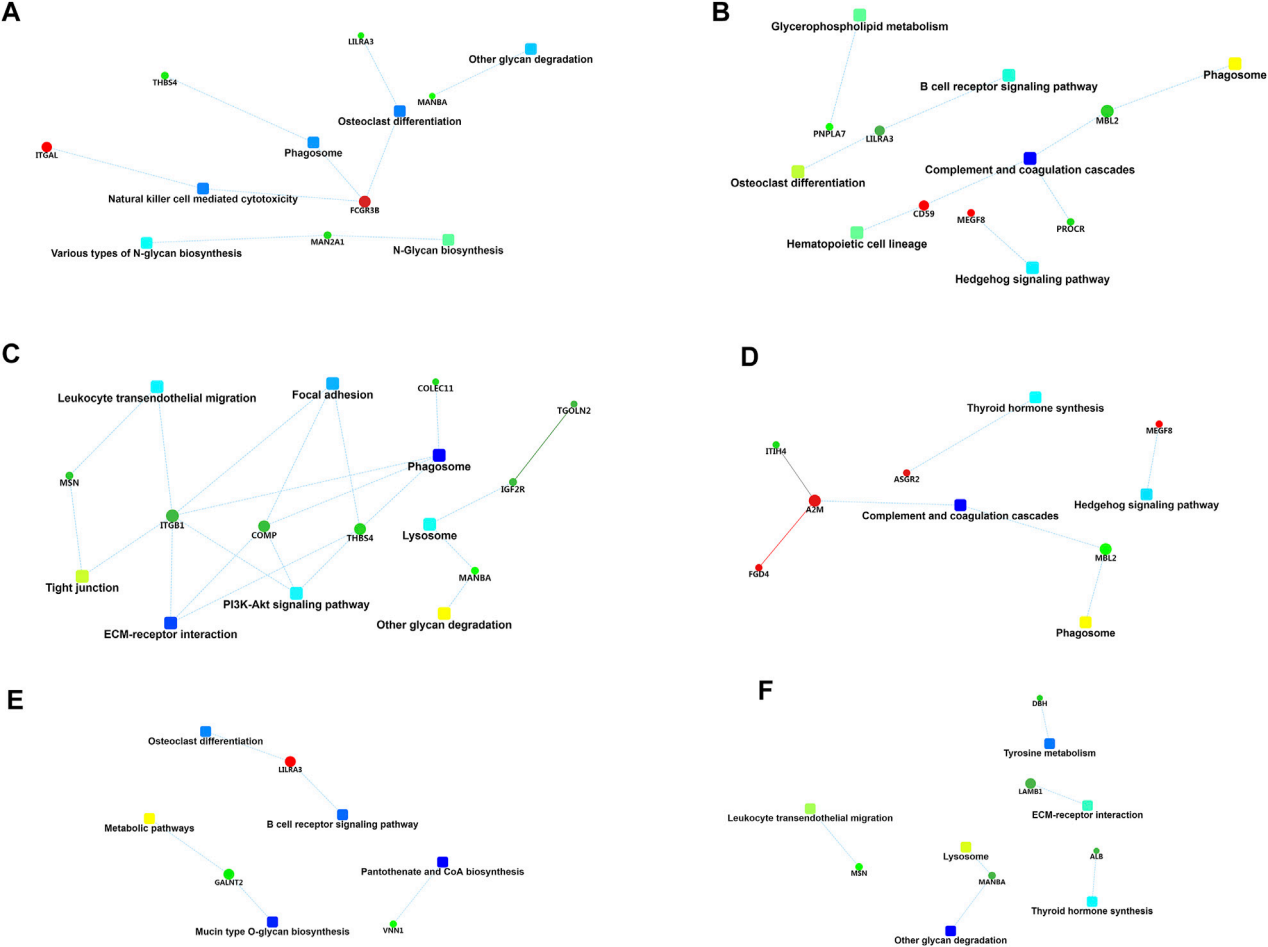
PPI network analysis of differentially expressed proteins: **(A)** group B (mild OSA) vs group A (non-OSA), **(B)** group C (moderate OSA) vs group A (non-OSA), **(C)** group D (severe OSA) vs group A (non-OSA), **(D)** group C (moderate OSA) vs group B (mild OSA), **(E)** group D (severe OSA) vs group B (mild OSA), and **(F)** group D (severe OSA) vs group C (moderate OSA).

The differentially expressed proteins between mild OSA and non-OSA groups were associated with natural killer-cell mediated cytotoxicity, phagosomes, osteoclast differentiation, and so on (Figure 8A). The differentially expressed proteins between moderate OSA and non-OSA groups were associated with glycerophospholipid metabolism, the B cell receptor signaling pathway, phagosomes, and so on (Figure 8B). The differentially expressed proteins between severe OSA and non-OSA groups were associated with leukocyte transendothelial migration, focal adhesion, phagosomes, and so on (Figure 8C). The differentially expressed proteins between moderate OSA and mild OSA groups were associated with the Hedgehog signaling pathway, complement and coagulation cascades, and phagosomes (Figure 8D). The differentially expressed proteins between severe OSA and mild OSA groups were associated with osteoclast differentiation, the B cell receptor signaling pathway, metabolic pathways, and so on (Figure 8E). The differentially expressed proteins between severe OSA and moderate OSA groups were associated with the leukocyte transendothelial migration, tyrosine metabolism, extracellular matrix (ECM)–receptor interaction, and so on (Figure 8F).

### Validation of Differentially Expressed Proteins in the Serum of OSA Patients as Determined by ELISA

**FIGURE 9:**
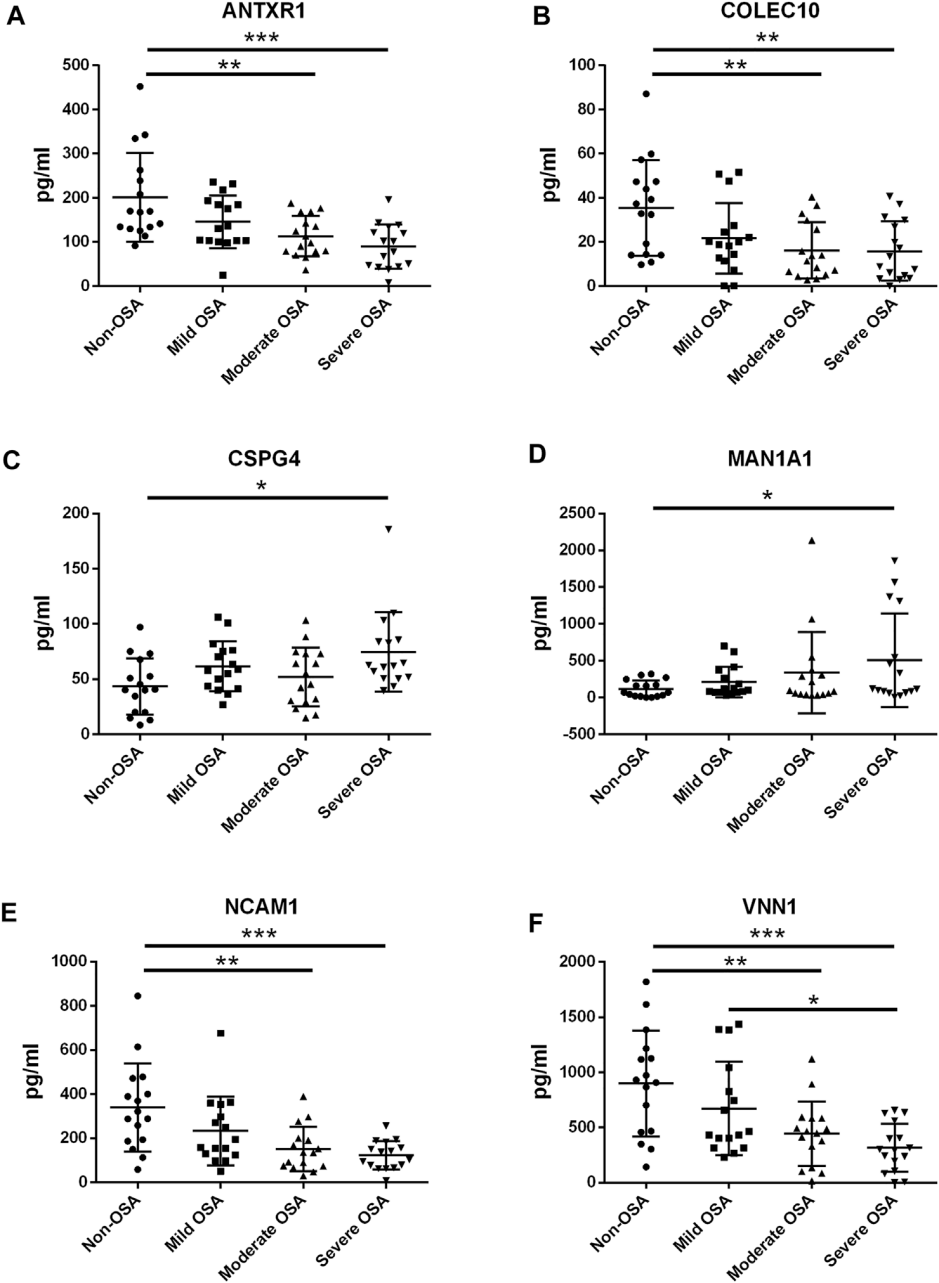
ELISA analysis of serum protein levels of ANTXR1 **(A)**, COLEC10 **(B)**, CSPG4 **(C)**, MAN1A1 **(D)**, NCAM1 **(E)**, and VNN1 **(F)** in the pediatric OSA. Significant differences between the treatment groups are indicated as *p < 0.05, **p < 0.01, and ***p < 0.001.

**FIGURE 10:**
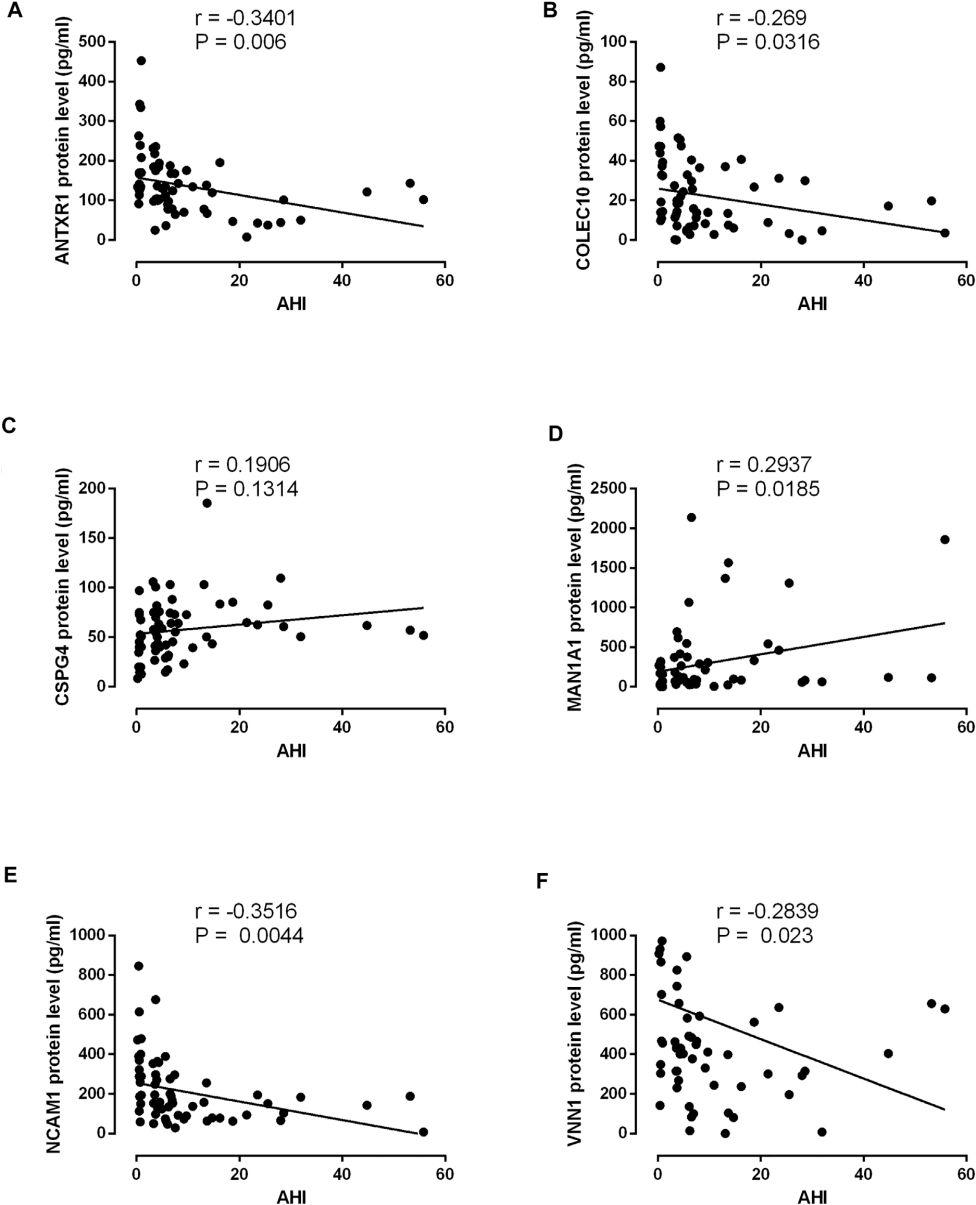
Correlation analysis between AHI and ANTXR1 **(A)**, COLEC10 **(B)**, CSPG4 **(C)**, MAN1A1 **(D)**, NCAM1 **(E)**, and VNN1 **(F)** in the 64 recruited subjects.

The protein expression levels of ANTR1, COLEC10, CSPG4, MAN1A1, NCAM1, and VNN1 in the serum levels from non-OSA and OSA patients were further determined by ELISA. The protein levels of ANTXR1, COLEC10, NCAM1, and VNN1 were significantly decreased in the moderate and severe OSA patients when compared to the non-OSA patients (Figures 9A,B,E,F); the VNN1 protein level was also higher in the severe OSA patients than in non-OSA patients (Figure 9F). The protein levels of CSPG4 and MAN1A1 were significantly increased in the severe OSA patients when compared to the non-OSA patients (Figures 9C,D). The correlation analysis showed that the protein levels of ANTXR1, COLEC10, NCAM1, and VNN1 were inversely correlated with AHI in the recruited subjects (Figures 10A,B,E,F), while the protein level of MAN1A1 was positively correlated with AHI (Figure 10D), and no significant correlation was detected between CSPG4 protein and AHI (Figure 10C).

## Discussion

Obstructive sleep apnea (OSA) is a common respiratory disorder with an estimated prevalence of 3–6% in the general population. However, the underlying pathophysiology of OSA remains unclear (Patel, 2019; Arnaud et al., 2020). Recently, proteomic analysis using high-resolution and high-throughput mass spectrometry has been widely used in the field of medical sciences. In the present study, TMT-based proteomic analysis was performed in the serum of patients with OSA. The proteomic analysis revealed a set of differentially expressed proteins that may be associated with the pathophysiology of OSA. The differentially expressed proteins in patients with OSA were enriched in pathways including phagosome and glycan synthesis/degradation, immune response, and the Hedgehog signaling pathway, indicating that such functions are key targets of OSA. Moreover, the experimental validation studies revealed that four proteins including ANTXR1, COLEC10, NCAM1, and VNN1 showed severity-dependent downregulations in expression. The present study for the first time performed the TMT-based proteomic analysis in the serum of OSA patients, and the present findings provided the novel insights into the potential roles of differentially expressed proteins in the pathophysiology of OSA.

So far, proteomic analysis in patients with OSA has been performed in various studies. Shah et al. performed proteomic analysis using surface-enhanced laser desorption/ionization time-of-flight mass spectrometry in the serum from children with OSA and identified three differentially expressed proteins that were associated with OSA (Shah et al., 2006). Krishna performed the proteomic analysis using 2D-PAGE and matrix-assisted laser desorption/ionization time-of-flight approaches in the urine of 11 children without OSA and 11 children with OSA, and the four differentially expressed proteins in OSA were identified (Krishna et al., 2006). Gozal et al. used the same technique to analyze the proteomics in the urine of children with OSA and found that four proteins in urine-based ELISA exhibited good accurate identification of OSA (Gozal et al., 2009). Jurado-Gamez et al. used the iTRAQ techniques to analyze the proteomics in the serum of adult patients with OSA and revealed that protein alterations in OSA were primarily associated with derangements in lipid and vascular metabolic pathways (Jurado-Gamez et al., 2012). Recently, Seetho et al. performed urinary proteomic profile analyses using capillary electrophoresis–mass spectrometry in OSA and non-OSA obese groups and found that the differences in urinary proteomic profiles were associated with increased metabolic syndrome in obese OSA subjects (Seetho et al., 2014). Kohli et al. performed the iTRAQ-based proteomic analysis coupled with ELISA validation and found that urinary EPCR and dermcidin may emerge as novel biomarkers for screening severe OSA patients (Kohli et al., 2019). In the present study, we performed the TMT-based proteomic analysis in the serum of OSA patients and identified the differential expressed proteins between different groups using pairwise comparison. In this study, we found that the differentially expressed proteins in patients with OSA were enriched in pathways including phagosome and glycan synthesis/degradation, immune response, and the Hedgehog signaling pathway, suggesting that DESPs enriched in these pathways may be associated with OSA.

In the experimental validation studies, we found that ANTXR1, COLEC10, NCAM1, and VNN1 proteins were downregulated and CSPG4 and MAN1A1 were upregulated in the serum from OSA patients when compared to the non-OSA patients. ANTXR1, also known as tumor endothelial marker 8 (TEM8), is a highly conserved transmembrane glycoprotein overexpressed on tumor vasculature. ANTXR1 has been found to be overexpressed in various types of cancers including breast, pancreatic, gastric, and colon cancer and can promote the entrance of anthrax toxin into cells (Rmali et al., 2007; Cryan and Rogers, 2011; Evans et al., 2018). Inhibition of ANTXR1 could repress the angiogenesis of tumor tissues (Chaudhary et al., 2012). Baek et al. also suggested the important role of ANTXR1 in the regulation of RANKL-induced osteoclast differentiation and functions (Baek et al., 2019). As OSA has been found to be associated with the endothelial dysfunction, our findings may suggest that the downregulated expression of ANTXR1 could be associated with the endothelial dysfunction of OSA patients.

COLEC10, also known as collectin liver 1, is a collectin protein in humans that is encoded by the COLEC10 gene. CL-L1 exhibited widespread tissue distribution with high and co-localized expression in secretory epithelia and mucosa (Hansen et al., 2018). In a cross-sectional cohort study of systemic lupus erythematosus (SLE), decreased serum levels of CL-L1 were associated with SLE (Troldborg et al., 2015). In addition, COLEC10 has been suggested to be associated with the innate immunity (Hansen et al., 2016). OSA is also a multicomponent disorder, with intermittent hypoxia (IH) as the main trigger for the associated cardiovascular and metabolic alterations. This IH induces several consequences such as hemodynamic, hormonometabolic, oxidative, and immuno-inflammatory alterations (Arnaud et al., 2009). Thus, the reduced levels of COLEC10 protein in the serum may be associated with the dysregulated immune-inflammatory responses in OSA.

NCAM1, as an important protein, is involved in cellular processes and in cell–cell interactions, and the dysregulation of this is associated with diverse diseases including cognitive dysfunction (Sullivan et al., 2007). In fact, several studies have found that OSA was associated with cognitive impairment (Gagnon et al., 2014). In addition, siRNA-mediated NCAM1 gene silencing suppressed oxidative stress in pre-eclampsia by inhibiting the p38MAPK signaling pathway (Zhang et al., 2019b). Based on the above evidence, the reduced level of NCMA1 in the serum may be related to the cognitive impairment and oxidative stress in OSA. VNN1 is a glycosylphosphatidyl inositol-anchored pantetheinase that is highly expressed in the liver, gut, and kidney. It can catalyze the hydrolysis of pantetheine into cysteamine and pantothenic acid (vitamin B5). Functional studies have suggested a role for VNN1 in oxidative stress, inflammation, and cell migration (Naquet et al., 2014; Schalkwijk and Jansen, 2014). However, the role of VNN1 in OSA has not been revealed yet. The present study showed that the downregulation of VNN1 in the serum of OSA patients was severity-dependent, and VNN1 may play a role in response to the oxidative stress and inflammation of patients with OSA. CSPG4 is a cell surface proteoglycan, considered as an ideal tumor-associated antigen, that is, an oncoantigen. CSPG4 plays a central role in the oncogenic pathways required for malignant progression and metastasis (Harrer et al., 2019). Provided that OSA is associated with the increased risk of cancer development, the elevated protein levels of CSPG4 could be linked to the cancer development in the OSA patients. MAN1A1 belongs to the GH47 Golgi mannosidase I subfamily, which cleaves α-1,2-bound mannose sugars from high-mannose glycans, resulting in 5-mannose glycans. This glycan structure is the substrate for various glycosyl transferases that catalyze the formation of complex branched tri- or tetra-antennary N-glycans. Dysregulation of MAN1A1 has been shown to be associated with the tumor progression in various types of cancers including breast cancer, ovarian cancer, cholangiocarcinoma, and liver cancer (Legler et al., 2018; Phoomak et al., 2018; Hamester et al., 2019; Park et al., 2020). Our results showed that the MAN1A1 protein level was elevated in the patients with OSA, suggesting that this protein alteration may be linked to the increased risk of cancer development in OSA patients.

The present study has several limitations. First, the sample size for the TMT-based proteomic analysis is low, and future studies may increase the sample size for the TMT-based proteomic analysis to reveal more significant findings. Second, in the validation study, we used only 16 samples in each group, and future studies may include more samples from multiple centers to consolidate the current findings. Third, the clinical significance of the verified proteins in OSA has not been fully deciphered in the present study, which should be considered as a future direction.

## Conclusion

In summary, the present study for the first time identified differentially expressed proteins in the serum from OSA patients with different severities by using TMT-based proteomic analysis. The functional enrichment studies suggested that several signaling pathways may be associated with the pathophysiology of OSA. The experimental validation results indicated that six proteins, including ANTXR1, COLEC10, NCAM1, VNN1, CGPG4, and MAN1A1, may play important roles in the pathophysiology of OSA, which still require further mechanistic investigation.

## Data Availability

The data presented in the study are deposited in the PRIDE repository, accession number PXD032734.
