# Supplementary material for: Serum Proteomic Analysis by Tandem Mass Tag-Based Quantitative Proteomics in Pediatric Obstructive Sleep Apnea
Source: Front Mol Biosci. 2022 Apr 11;9:762336. doi: 10.3389/fmolb.2022.762336 (PMC9035643; doi:10.3389/fmolb.2022.762336)
Supplement: Supplementary file 2 [file Table1.DOCX]

Table 1. Clinical characteristics of all the recruited subjects

|  | Non-OSA | Mild OSA | Moderate OSA | Severe OSA |
| --- | --- | --- | --- | --- |
|  | (n =16) | (n = 16) | (n = 16) | (n = 16) |
| Age (years old) | 5.9 (2-11) | 4.8 (3–8) | 5.2 (2-13) | 5.4 (2–9) |
| BMI (kg/m2) | 16.2 (13.6-20.6) | 16.8 (13-29.7) | 16.3 (13.2–20.9) | 17.2 (14.5–21.5) |
| Awake SpO2 (%) | 98 (97–100) | 98 (97–100) | 98 (97–99) | 97 (96–98) |
| AHI | 0.62 (0.2–0.9) | 3.9 (3.2–4.9) | 6.9 (5.6–9.2) | 25.9 (10.9–55.8) |
| SpO2 minimum (%) | 92 (87–96) | 90 (83–93) | 83 (72–90) | 76 (51–88) |
| SpO2 mean (%) | 98 (96–100) | 98 (96–100) | 98 (96–99) | 96 (92–98) |
