# Supplementary material for: Serum Proteomic Analysis by Tandem Mass Tag-Based Quantitative Proteomics in Pediatric Obstructive Sleep Apnea
Source: Front Mol Biosci. 2022 Apr 11;9:762336. doi: 10.3389/fmolb.2022.762336 (PMC9035643; doi:10.3389/fmolb.2022.762336)
Supplement: Supplementary file 3 [file Table2.DOCX]

Supplemental Table S1. Clinical characteristics of patients for TMT-based proteomic analysis

|  | Non-OSA | Mild OSA | Moderate OSA | Severe OSA |
| --- | --- | --- | --- | --- |
|  | (n =3) | (n = 3) | (n = 3) | (n = 3) |
| Age (years old) | 5.3 (3-7) | 4.7 (3–7) | 3.7 (2-5) | 7.3 (6–9) |
| BMI (kg/m2) | 16.7 (14.9-18.4) | 14.3 (13-15.3) | 16.5 (13.6–18.3) | 18.4 (21.4–28.6) |
| Awake SpO2 (%) | 98 (96–99) | 98.2 (96–100) | 97.3 (96–99) | 96.7 (96–97) |
| AHI | 0.47 (0.4–0.5) | 4.4 (4.0–4.9) | 7.7 (7.4–8.1) | 24.5(21.4–28.6) |
| SpO2 minimum (%) | 92.3 (91–93) | 90.7 (90–91) | 81 (72–86) | 77 (69–86) |
| SpO2 mean (%) | 98 (96–99) | 97.3 (96–98) | 97 (96–98) | 96 (96–96) |
