# Supplementary material for: Serum Proteomic Analysis by Tandem Mass Tag-Based Quantitative Proteomics in Pediatric Obstructive Sleep Apnea
Source: Front Mol Biosci. 2022 Apr 11;9:762336. doi: 10.3389/fmolb.2022.762336 (PMC9035643; doi:10.3389/fmolb.2022.762336)
Supplement: Supplementary file 4 [file Table4.DOCX]

**Serum proteomic analysis by tandem mass tags based quantitative proteomics in pediatric obstructive sleep apnea**

Hanrong Cheng^1,#^ (Email: chenghanrongsz@163.com)

Shoumei Jin^2,#^ (Email: 41143679@qq.com)

Simin Huang^2^ (Email: huangsiminsimin@163.com)

Tianyong Hu^2^ (Email: 402995674@qq.com)

Miao Zhao^2^ (Email: bondshine@qq.com)

Dongcai Li^2,*^ (Email: dongcailisz@163.com)

Benqing Wu^3,*^ (Email: wubenqing783@126.com)

^1^Institute of Respiratory Diseases, Shenzhen People’s Hospital, The Second Clinical Medical College of Jinan University, The First Affiliated Hospital of Southern University of Science and Technology, Shenzhen, Guangdong, 518020, China

^2^Longgang ENT Hospital, Institute of ENT and Shenzhen Key Laboratory of ENT, Shenzhen, Guangdong, 518172, China

^3^Department of Neonatology, University of Chinese Academy of Science-Shenzhen Hospital (Guangming), Shenzhen, Guangdong, 518000, China

*Corresponding authors: Dongcai Li and Benqing Wu


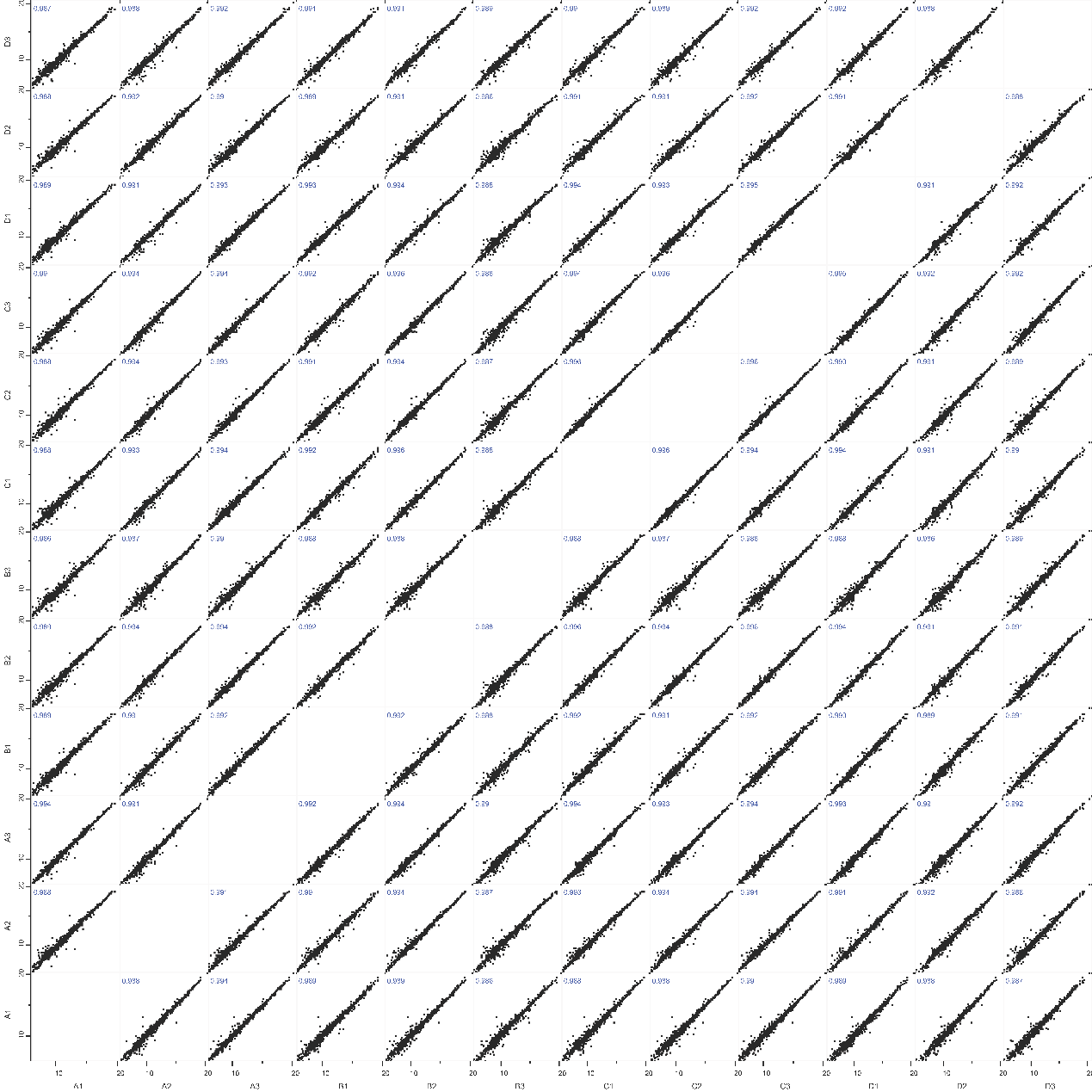


**Supplemental Figure S1.** A matrix of scatter plots and Pearson correlation coefficient of protein intensities for each sample.
